# A Traditional Chinese Medicine Xiao-Ai-Tong Suppresses Pain through Modulation of Cytokines and Prevents Adverse Reactions of Morphine Treatment in Bone Cancer Pain Patients

**DOI:** 10.1155/2015/961635

**Published:** 2015-11-04

**Authors:** Yan Cong, Kefu Sun, Xueming He, Jinxuan Li, Yanbin Dong, Bin Zheng, Xiao Tan, Xue-Jun Song

**Affiliations:** ^1^Center for Clinical Research and Translational Medicine, Lianyungang Oriental Hospital, Lianyungang, Jiangsu 222042, China; ^2^Department of Traditional Chinese and Western Medicine, Lianyungang Oriental Hospital, Lianyungang, Jiangsu 222042, China; ^3^Department of Orthopedics, The First Affiliated Hospital of Soochow University, Suzhou 215006, China

## Abstract

Treating cancer pain continues to possess a major challenge. Here, we report that a traditional Chinese medicine Xiao-Ai-Tong (XAT) can effectively suppress pain and adverse reactions following morphine treatment in patients with bone cancer pain. Visual Analogue Scale (VAS) and Quality of Life Questionnaire (EORTC QLQ-C30) were used for patient's self-evaluation of pain intensity and evaluating changes of adverse reactions including constipation, nausea, fatigue, and anorexia, respectively, before and after treatment prescriptions. The clinical trials showed that repetitive oral administration of XAT (200 mL, bid, for 7 consecutive days) alone greatly reduced cancer pain. Repetitive treatment with a combination of XAT and morphine (20 mg and 30 mg, resp.) produced significant synergistic analgesic effects. Meanwhile, XAT greatly reduced the adverse reactions associated with cancer and/or morphine treatment. In addition, XAT treatment significantly reduced the proinflammatory cytokines interleukin-1*β* and tumor necrosis factor-*α* and increased the endogenous anti-inflammatory cytokine interleukin-10 in blood. These findings demonstrate that XAT can effectively reduce bone cancer pain probably mediated by the cytokine mechanisms, facilitate analgesic effect of morphine, and prevent or reduce the associated adverse reactions, supporting a use of XAT, alone or with morphine, in treating bone cancer pain in clinic.

## 1. Introduction

Pain is a common symptom in patients with advanced malignant cancer and greatly increases physical and mental suffering. There are approximately ten million new cancer patients annually worldwide. Cancer pain is observed in 30%–50% of all these new-onset patients. Most of the patients (60%–90%) with advanced cancer present different degrees of pain, of which 30% are with unbearable pain symptoms [1, 2]. The pathophysiology of cancer pain remains elusive and therapeutic approaches are very limited. Surgery, radiotherapy, and chemotherapy have been used as effective approaches to treating certain cancer pain, but pain in more than half of the cancer patients remains unrelieved [3]. Relieving cancer pain is critical to improving quality of life of the cancer patients. The “Three-Step Therapy” for treating cancer pain proposed by World Health Organization (WHO) thirty years ago has been widely implemented clinically and accepted by a majority of cancer patients and medical professionals, but the clinical effectiveness is still limited. Continuous efforts are needed to realize the goal of “relieving pain of all cancer patients.” To effectively, safely, and economically control pain and to improve the quality of life for the patients with cancer pain are important issues of global concern.

Traditional Chinese medicine is an alternative, effective therapeutic measure for treating cancer pain and has potential to overcome the shortcomings of the “Three-Step Therapy” by enhancing treatment effect and reducing toxicity of morphine, the core drug used in the WHO “Three-Step Therapy” [4]. A traditional Chinese medicine named Xiao-Ai-Tong (XAT), a prescription medication (decoctio) in Chinese, consists of Corydalis Tuber (tuber of* Corydalis* turtschaninovii,* yanhusuo*), wild ginger (*Asarum*),* Arisaema consanguineum*, Venenum Bufonis, and Arisaema Tuber (Tian-Nan-Xing, Rhizoma Arisaematis) and has been used in clinic for years for reducing chronic pain. However, there is no evidence-based research demonstrating analgesic effect of XAT on cancer pain patients. This clinical study aimed to evaluate the analgesic effect of XAT and its impact on morphine treatment-induced adverse reactions including abstraction, nausea/vomiting, fatigue, and anorexia and quality of life in patients with moderate-to-severe cancer pain. Our results indicate that XAT decoction can effectively alleviate bone cancer pain, suppress morphine-induced adverse actions, reduce the proinflammatory cytokines IL-1*β* and TNF-*α*, and increase the endogenous anti-inflammatory cytokine IL-10. This study supports a clinical use of XAT, alone or with morphine, in treating bone cancer pain.

## 2. Materials and Methods

### 2.1. Patient General Information

Patients with painful bone metastasis received treatment at Lianyungang City Oriental Hospital. The clinical trials were conducted according to Declaration of Helsinki principles, approved by the ethics committee of Lianyungang Oriental Hospital (number 20130910088) (2013). A total of 60 patients with painful bone metastasis were included in this study including 32 males (53.33%) and 28 females (46.67%), aged from 44 to 70 years (mean ± SD, 58.36 ± 7.31). These patients were randomly divided into four groups: (1) firstly, based on the time sequence, the 1st, 2nd, 3rd, and 4th patient recruited were included in groups 1, 2, 3, and 4, respectively. Subsequently, the 5th to 8th patients recruited were included in groups 1, 2, 3, and 4 again, respectively. Then this method of grouping was repeated for all the patients that were recruited later for this study. However, considering balance and similar proportion of male and female patients in each of the groups, the male and female patients were grouped in a separate time sequence in the same way as in the different groups. Four groups received four different treatments (see treatment procedures in Section 2.2). Each of the four groups included 15 patients with 8 males and 7 females.

Inclusion criteria for these patients were as follows: (1) pathologically diagnosed as cancer accompanied by painful osseous metastasis, requiring drug treatment; (2) with normal pain sensation and judgment (without intellectual or mental disorders); (3) the patient's pain self-evaluation Visual Analogue Scales (VAS) [5, 6] test score reaching 4 or higher; and (4) expected survival being longer than one month.

Exclusion criteria for these patients were as follows: (1) with dysfunctions of heart, liver, kidney, and other severe physical disorders; (2) with histories of psychiatric or analgesic drug abuse; (3) with history of significant respiratory depression, airway obstruction, hypoxia, or bronchial asthma; or (4) received radiotherapy and/or chemotherapy, and so forth.

### 2.2. Treatment Prescriptions and Procedures

Patients that received morphine treatment were taking morphine sulfate controlled-release tablets (each 10 mg tablet provided by Beijing Mundipharma Pharmaceutical Co., Ltd.), 20 or 30 mg, q12h, by oral administration for 7 consecutive days. Patients who received XAT treatment were taking XAT decoction containing Corydalis Tuber, wild ginger,* Arisaema consanguineum*, Venenum Bufonis, and Arisaema Tuber, 200 mL, q12h, by oral administration for 7 consecutive days. Two groups of patients received treatment of XAT (200 mL) + morphine (20 mg and 30 mg, resp., q12h, by oral administration for 7 consecutive days). The seven treatments in each group were applied daily during days 0–6.

### 2.3. Evaluation of Pain Intensity

Changes in pain intensity were evaluated by calculating changes of VAS scores [5, 6], which was used for patient's self-evaluation. VAS scores were ranging from 0 to 10 marked on a horizontal line, where 0 represented painless condition, 1–3 referred to mild pain, 4–6 denoted moderate pain, and 7–10 suggested severe pain. At the testing moment, the patients were asked to circle a number that best represented their pain. VAS test was given at 3 and 1 days prior to the treatment and once a day during 1–7 postoperative days following the 7 treatments during days 0–6. Two more tests were given on the 10th and 14th day after termination of the treatments.

### 2.4. Adverse Reactions due to Morphine Treatment

The in-patients sometimes showed adverse reactions such as expiratory dyspnea, changes in blood pressure, pale complexion, cold sweat, palpitation, headache, dizziness, lethargy, delirium, blurred vision, instability of gait, dryness, nausea, vomiting, constipation, bellyache, diarrhea, pruritus of skin, skin rashes, and dysuria. Morphine treatment resulted in or facilitated some of these adverse reactions. These signs were honestly recorded in each of the in-patient's profiles, but most of them were not included in the data show in this study. The data we monitored and showed here included four symptoms, nausea with or without vomiting, constipation, anorexia, and fatigue, which are expressed based on the analysis using the Quality of Life Questionnaire (EORTC QLQ-C30) [7]. This EORTC's scores were used for evaluating the changes of these adverse reactions and was given on day 7 and day 10, that is, 1–4 days after termination of the treatment.

### 2.5. Statistical Analysis

SPSS Rel 17 was used to conduct all the statistical analyses. Alterations of pain intensity following treatment over time among groups were tested with two-way ANOVA with repeated measures followed by Bonferroni post hoc tests, respectively. Differences in changes of the aversive reactions among groups were tested with one-way ANOVA. Data are presented as mean ± SEM with VAS tests or EORTC QLQ-C30 scores, except when indicated separately. Statistical results are considered significant if *P* < 0.05.

## 3. Results

The total of 60 patients with painful bone metastasis were included in this study including 32 males (53.33%) and 28 females (46.67%), aged from 44 to 70 years (mean ± SD, 58.65 ± 7.38). These patients were randomly divided into four groups (male and female patients were kept at similar proportion in each group). Each of the groups received different treatments and included 15 patients with 8 males and 7 females. Patients who received morphine treatment (morphine group) included 15 patients with 8 males aged 58.10 ± 7.14 and 7 females aged 58.04 ± 8.26. Patients who received XAT treatment (XAT group) included 15 patients with 8 males aged 57.60 ± 8.14 and 7 females aged 59.40 ± 7.09. Two groups of patients who received treatment of combination of XAT and morphine (in two different doses) included, in each group, 15 patients with 8 males aged 58.22 ± 8.45 and 57.15 ± 7.62 and 7 females aged 59.33 ± 6.53 and 58.87 ± 6.76, respectively. The data included here were from these 60 patients.

### 3.1. Repetitive Oral Administration of XAT Reduced Cancer Pain and Facilitated Analgesic Effect of Morphine

**Figure 1:**
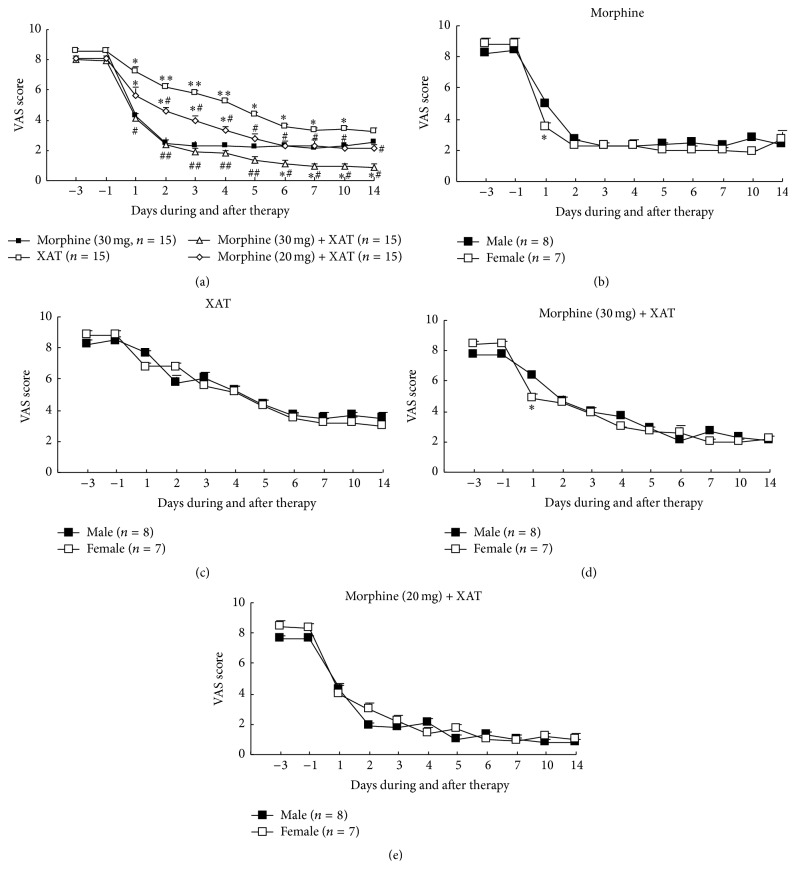
Repetitive oral administration of XAT reduced cancer pain and facilitated analgesic effect of morphine on patients with bone cancer pain. VAS test was used for evaluating the pain intensity. (a) Comparison of analgesic effects (VAS scores) of morphine, XAT, and combination of XAT and morphine. (b-c) Comparison of analgesic effects of morphine, XAT, and combination of XAT and morphine between males and females. Data are expressed as mean ± SEM. Number of patients in each group is indicated in the parentheses. Note that all of the patients indicated in (b-c) were from the same male and female patients' group in (a). The treatments in each group were applied daily for 7 consecutive days during days 0–6. Two-way ANOVA. ^*∗*^ *P* < 0.05 and ^*∗∗*^ *P* < 0.01 versus morphine (a) or versus male (b, d). ^#^ *P* < 0.05 and ^##^ *P* < 0.01 versus XAT (a).

Patients in this study received treatment of morphine, XAT, and morphine + XAT, respectively. As expected, morphine treatment alone (30 mg, every 12 h, and oral administration for 7 consecutive days) greatly reduced cancer pain evidenced by the significantly decreased VAS scores. The analgesic effect quickly reached the peak level after the second dose and was maintained at this level for up to 14 days without obvious tolerance. Interestingly, repetitive oral administration of the Chinese medicine XAT (200 mL, every 12 h, and oral administration for 7 consecutive days) produced significant, time-dependent, gradually increased analgesic effect on the cancer pain. The significant analgesic effect was starting from the second day (XAT) and reached peak 5-6 days after the daily treatment. The peak analgesic effect was maintained for up to at least 14 days, that is, 8 days after termination of the last treatment. Analgesic effect of XAT was significantly less than that of morphine during the period of 1–5 days. However, during 6–14 days, the analgesic effect of XAT alone was much closer to that of morphine alone. Furthermore, administration of a combination of XAT with morphine at 20 mg produced significant analgesic effect, which was at the similar level of analgesia produced by morphine alone at 30 mg. Administration of a combination of XAT with morphine at 30 mg produced significantly greater analgesic effect than that produced by morphine alone at 30 mg, XAT with morphine at 20 mg, and XAT alone. These analgesic effects were maintained as the best level of analgesia for at least one week after termination of the last treatment. Data are summarized in Figure 1(a).

We also analyzed the possible sexual difference of analgesic effects of XAT and morphine on these patients (Figures 1(b)–1(d)). The data was from the same group of the patients in Figure 1(a). The data point at day 1 was as follows: the VAS scores for female patients in the groups of morphine treatment (30 mg) (Figure 1(b)) and morphine (30 mg) with XAT treatment (Figure 1(d)) were significantly lower than that of male patients, suggesting that these females had better response to the first-time analgesic treatment. However, the analgesic effects of XAT and morphine and their combinations were overall not significantly different between male and female patients (Figures 1(b)–1(d)).

### 3.2. Repetitive Oral Administration of XAT Reduced or Prevented Morphine-Induced Adverse Reactions

**Figure 2:**
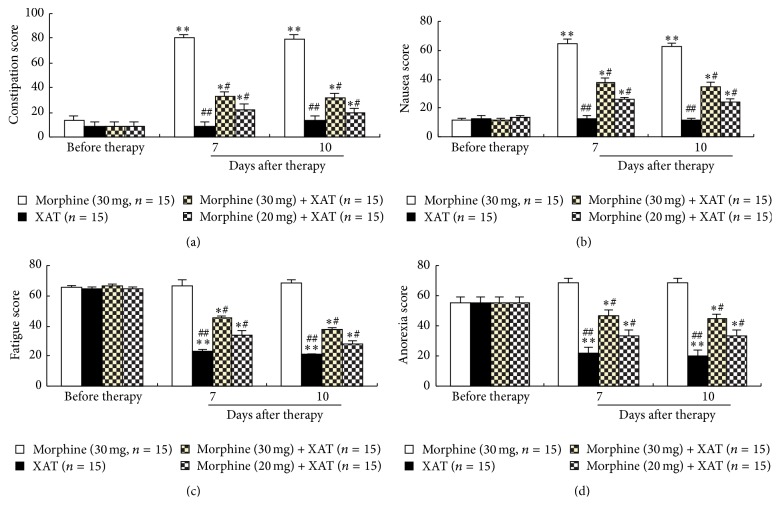
Effects of repetitive oral administration of XAT on the adverse reactions accompanied with cancer pain and/or morphine treatment. The Quality of Life Questionnaire (EORTC QLQ-C30) was used to score the adverse reactions. The higher the score, the worse the adverse reaction. These adverse reactions included constipation (a), nausea (b), fatigue (c), and anorexia (d). Comparisons were made among groups of morphine, XAT, and combination of XAT and morphine at two different doses. Data are expressed as mean ± SEM. Number of patients in each group is indicated in the parentheses and all of the patients indicated in (a–d) were the same patients included in Figure 1(a) and they received the same treatments in the same protocols in the corresponding groups. The evaluation was made on day 7 and day 10, that is, 1 and 4 days after termination of the last treatment. One-way ANOVA. ^*∗*^ *P* < 0.05 and ^*∗∗*^ *P* < 0.01 versus the same group before therapy. ^#^ *P* < 0.05 and ^##^ *P* < 0.01 versus morphine after therapy in the corresponding group.

Given that repetitive oral administration of XAT reduced cancer pain and facilitated analgesic effect of morphine, we continued to analyze the possible effects of the Chinese medicine XAT on the adverse reactions accompanied with cancer pain and/or with morphine treatment. We used the Quality of Life Questionnaire (EORTC QLQ-C30) to score the adverse reactions in these patients. The higher the score, the worse the adverse reaction. The data showed that the scores of the gastrointestinal adverse reactions constipation and nausea were greatly increased after repetitive morphine treatment (morphine 30 mg, daily for 7 consecutive days). These morphine treatment-related adverse reactions were significantly prevented or reduced by the treatment of a combination of XAT with morphine (Figures 2(a) and 2(b)). Another two adverse symptoms, fatigue and anorexia, were found in all of these cancer pain patients before application of our treatment prescription, indicating that these two symptoms were because of the cancer and/or cancer pain. The scores of fatigue and anorexia were greatly reduced by repetitive XAT treatment, but not by morphine (Figures 2(c) and 2(d)).

### 3.3. Repetitive Oral Administration of XAT Inhibited Proinflammatory Cytokines IL-1*β* and TNF-*α* and Activated IL-10

**Figure 3:**
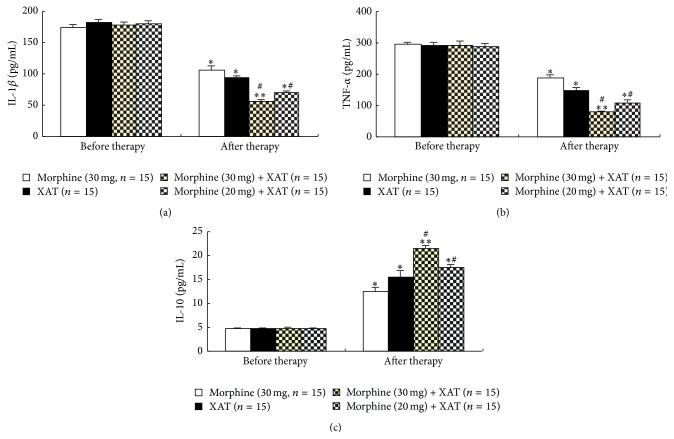
Effects of repetitive oral administration of XAT, morphine, and their combinations on the blood cytokines IL-1*β*, TNF-*α*, and IL-10. ELISA was used to measure these cytokines in blood from the cancer pain patients. Number of patients in each group is indicated in the parentheses and all of these patients were the same patients included in Figure 1(a) and they received the same treatments in the same protocols in the corresponding groups. The blood samples were collected on day 7, that is, 1 day after termination of the last treatment. One-way ANOVA. ^*∗*^ *P* < 0.05 and ^*∗∗*^ *P* < 0.01 versus the same group before therapy. ^#^ *P* < 0.05 versus morphine after therapy in the corresponding group.

We continued to examine whether the Chinese medicine XAT might affect the activity of the proinflammatory cytokines IL-1*β* and TNF-*α*, which are important in the development of inflammation and cancer pain, as well as the anti-inflammatory cytokine IL-10, which may inhibit inflammation and pain. Levels of these three cytokines in blood in these patients before application of our treatment prescriptions were 174 ± 25 pg/mL (IL-1*β*, Figure 3(a)), 297 ± 35 pg/mL (TNF-*α*, Figure 3(b)), and 4.70 ± 0.79 pg/mL (IL-10, Figure 3(c)), respectively. Repetitive treatment of XAT, morphine, or combinations of XAT and morphine, in the same protocols described above, greatly reduced the levels of IL-1*β* and TNF-*α*. Meanwhile, level of IL-10 was greatly increased following the treatments. Among these four different therapies, the combination of XAT and morphine produced the best modulatory effects on the three cytokines. Data are summarized in Figure 3.

## 4. Discussion

This study reveals that repetitive oral administration of the Chinese traditional medicine XAT decoction can suppress pain probably through modulation of cytokines and prevents adverse reactions of morphine treatment in bone cancer pain patients. The major findings are fourfold: (1) repetitive oral administration of XAT for 7 consecutive days greatly reduces bone cancer pain; (2) treatment with combination of XAT and morphine produces synergistic analgesic effects; (3) XAT can prevent or alleviate the gastrointestinal adverse reactions constipation, nausea, fatigue, and anorexia accompanied with cancer pain and/or caused by morphine treatment; and (4) XAT treatment significantly reduces the proinflammatory cytokines IL-1*β* and TBF-*α* and increases the endogenous anti-inflammatory cytokine IL-10 in blood. These results demonstrate that XAT has a strong capacity of relieving bone cancer pain probably at least partly through its modulation of cytokines in patients as well as a capacity of antigastrointestinal adverse reactions, caused by cancer/cancer pain and/or morphine treatment. This study supports a use of XAT decoction in treating bone cancer pain, alone or with morphine, in clinic.

Our clinical trials indicate that the Chinese medicine XAT decoction can be used as an effective analgesic for treating bone cancer pain and the associated adverse reactions such as fatigue and anorexia in cancer patients. Further, if used together with morphine, XAT can facilitate the analgesic effect and reduce the side effects including constipation and nausea caused by morphine treatment. This study supports that XAT may be a good candidate for being used alone or with morphine in treating cancer pain in clinic and suggests a general idea that an integrated treatment measure combining certain Chinese herbs or other therapeutic measures such as acupuncture with opioids such as morphine may be a great strategy for treating cancer pain and thus improve the treatment effect of WHO “Three-Step Therapy” for cancer pain. It is an ideal strategy to have effective pain relief with minimal side effects, which greatly enhances quality of life of the patients, particularly for the severe terminal cancer patients.

Cancer pain is a severe, lifelong, intractable chronic pain [1–3]. Pathophysiology of cancer pain is very complicated and involved at least in two complex mechanisms, that is, neuropathic and inflammatory pain mechanisms [3, 8, 9]. Studies have confirmed that bone cancer pain is associated with the tumor itself, primary or metastatic bone cancer [8–10], and changes in the local bone tissues [11, 12]. Bone cancer pain may be initiated by compression and stimulation of the neighboring sensory nerves by tumor tissues, tumor cell, and related immune cell released cytokines, which sensitize the nociceptors, bone deconstruction, and local ischemia as well as hypoxic microenvironment caused by the tumor [13, 14]. All of these elements contribute greatly to the development of cancer pain. The complex mechanisms of cancer pain result in a big trouble to well control the cancer pain in clinic.

When we are pursuing better strategy to treat cancer pain, some of the Chinese medicines may provide us with a different view. According to traditional Chinese medicine, the primary pathogenesis of cancer pain involves “stagnation of pneuma and blood” or “malnutrition.” “Malnutrition” is primarily attributed to renal deficiency unlikely to promote bone marrow generation. “Stagnation of pneuma and blood” is mainly associated with the Qi stagnation due to severe cold and blood stasis [2]. Cancer pain has been brilliantly described in ancient medical books of traditional Chinese medicine. It was described in* The Medicine of The Yellow Emperor* as “bones becoming dry and brittle, muscles becoming emaciated, thoracic fullness, asthmatic inconvenience, inner pain inducing shoulder and neck pain.” Based on the idea of the traditional Chinese medicine, we believe that the cancer pain may be caused by accumulation of toxicity, blood stasis and vital energy retardation, and accumulation of phlegm-dampness in the meridians, and thus resulting in pneuma and blood stasis, which contribute to development of cancer pain. The therapeutic effects of traditional Chinese medicines, in a concept, have been confirmed, as persistent, nonadditive and nonresistant drugs. Some analgesic herbs can be used alone to treat cancer pain in the first and second steps of the WHO “Three-Step Therapy” and considered as a preferred combination in the third step of the “Three-Step Therapy” to reduce the dose of opioid analgesics and the toxicity. In this study, we recommend that XAT decoction, which contains Corydalis Tuber, wild ginger,* Arisaema consanguineum*, Venenum Bufonis, and Arisaema Tuber, may be used in clinic in treating cancer pain. Actually, recent studies have shown that certain Chinese herbs such as the Corydalis Rhizoma, named Yuanhu in Chinese, have been proved to have analgesic effect on patients and on small animals with neuropathic pain as well as other treatment effects [15–19]. Our clinical observations here demonstrate that XAT, containing Corydalis Rhizoma and so forth, used alone and with combination of morphine, has a great value for treating bone cancer pain in patients. XAT may reduce cancer pain, reduce doses of morphine, and diminish the adverse reactions caused by morphine.

Studies have demonstrated that astrocytes and microglial cells, which act as parts of the innate immune system, become active in the status of bone cancer and release various substances, including the proinflammatory cytokines IL-1*β* and TNF-*α*, which could evoke hyperalgesia and allodynia [20–25]. In this study, we show that repetitive oral administration of XAT decoction for a week can greatly suppress the increased IL-1*β* and TNF-*α* associated with the cancer pain. Meanwhile, XAT therapy significantly increases the anti-inflammatory cytokines IL-10. These findings may provide an explanation for the analgesic effect of XAT. Further large-scale clinical studies as well as studies in small animals for underlying the possible mechanisms of analgesia of XAT are urgently needed.

## 5. Conclusions

This clinical trial indicates that the Chinese medicine XAT decoction is an effective analgesic for treating chronic pain and can alleviate certain adverse reactions such as fatigue and anorexia associated with bone cancer pain and, if used with morphine, can prevent or reduce repetitive morphine treatment-induced constipation and nausea. This study supports a clinical use of XAT decoction in patients with bone cancer pain and proposes a general idea that a combination of certain Chinese herbs with opioids may be a better strategy for treating cancer pain and the morphine-treatment associated adverse reactions, thus improving the treatment effect by the WHO “Three-Step Therapy” for cancer pain. It is an ideal strategy to have effective pain relief with minimal side effects that greatly enhances quality of life of the patients, particularly for the terminal cancer patients with severe pain.
